# Disruption of Boundary Encoding During Sensorimotor Sequence Learning: An MEG Study

**DOI:** 10.3389/fnhum.2018.00240

**Published:** 2018-06-12

**Authors:** Georgios Michail, Vadim V. Nikulin, Gabriel Curio, Burkhard Maess, María Herrojo Ruiz

**Affiliations:** ^1^Neurophysics Group, Department of Neurology, Campus Benjamin Franklin, Charité—Universitätsmedizin Berlin, Berlin, Germany; ^2^Department of Psychiatry and Psychotherapy, St. Hedwig Hospital, Charité—Universitätsmedizin Berlin, Berlin, Germany; ^3^Department of Neurology, Max Planck Institute for Human Cognitive and Brain Sciences, Leipzig, Germany; ^4^Center for Cognition and Decision Making, National Research University Higher School of Economics, Moscow, Russia; ^5^Research Group “MEG and Cortical Networks”, Max Planck Institute for Human Cognitive and Brain Sciences, Leipzig, Germany; ^6^Department of Psychology, Whitehead Building, Goldsmiths, University of London, London, United Kingdom

**Keywords:** serial order, boundaries, prefrontal cortex, supplementary motor area, sensorimotor learning, sequence learning

## Abstract

Music performance relies on the ability to learn and execute actions and their associated sounds. The process of learning these auditory-motor contingencies depends on the proper encoding of the serial order of the actions and sounds. Among the different serial positions of a behavioral sequence, the first and last (boundary) elements are particularly relevant. Animal and patient studies have demonstrated a specific neural representation for boundary elements in prefrontal cortical regions and in the basal ganglia, highlighting the relevance of their proper encoding. The neural mechanisms underlying the encoding of sequence boundaries in the general human population remain, however, largely unknown. In this study, we examined how alterations of auditory feedback, introduced at different ordinal positions (boundary or within-sequence element), affect the neural and behavioral responses during sensorimotor sequence learning. Analysing the neuromagnetic signals from 20 participants while they performed short piano sequences under the occasional effect of altered feedback (AF), we found that at around 150–200 ms post-keystroke, the neural activities in the dorsolateral prefrontal cortex (DLPFC) and supplementary motor area (SMA) were dissociated for boundary and within-sequence elements. Furthermore, the behavioral data demonstrated that feedback alterations on boundaries led to greater performance costs, such as more errors in the subsequent keystrokes. These findings jointly support the idea that the proper encoding of boundaries is critical in acquiring sensorimotor sequences. They also provide evidence for the involvement of a distinct neural circuitry in humans including prefrontal and higher-order motor areas during the encoding of the different classes of serial order.

## Introduction

A broad spectrum of daily tasks, such as preparing a meal or washing hands, requires the learning and production of sequential movements. These processes also support more complex forms of sensorimotor behavior, such as speech or music performance, which additionally require the processing of auditory feedback to control the production of motor output. The unique demands that music performance (including singing) poses on the underlying neural circuitry—namely, higher precision in temporal (i.e., rhythm) and spectral properties (i.e., pitch) than speech—make it a useful model for investigating the neural mechanisms at the base of sensorimotor sequence learning (Natke et al., 2003; Zatorre et al., 2007; Herrojo Ruiz et al., 2009, 2011; Patel, 2011; Zatorre and Baum, 2012).

Sequence learning requires organizing single actions in a specific temporal *serial order* to build a larger action unit. A large body of evidence suggests the involvement of frontoparietal, basal ganglia, and cerebellar circuits during sequence learning (Mushiake and Strick, 1995; Hikosaka et al., 1999; Averbeck et al., 2002; Fujii and Graybiel, 2003; Kao et al., 2005; Lehéricy et al., 2005; Penhune and Doyon, 2005; Ölveczky et al., 2011; Wymbs et al., 2012). Also, a neural representation of serial order coding has been reported in primate frontal areas—the prefrontal cortex (PFC), supplementary motor area (SMA) and primary motor cortex (M1)—as well as primate and rodent striatal areas (Tanji and Shima, 1994; Procyk and Joseph, 2001; Averbeck et al., 2002; Fujii and Graybiel, 2005; Lu and Ashe, 2005).

Animal studies suggest that in addition to the encoding of serial order, the encoding of boundary elements at the beginning and the end of the sequence is crucial for the acquisition of motor sequences (Fujii and Graybiel, 2003, 2005; Jin and Costa, 2010). In these studies, neuronal ensembles in the PFC and basal ganglia nuclei showed an increased neural response at the boundary elements (“*Bo*”) of a sequential task. Another study found that in macaque’s PFC, boundaries were associated with stronger neural representations compared to within-sequence (“*In*”) elements (Averbeck et al., 2002).

Crucially, the findings in non-human animals relating to specific neural representations for first and last sequence elements emphasize the relevance of sequence boundaries during sequence learning, as was also postulated in theoretical models of serial order memory (Dehaene and Changeux, 1997; Henson, 1998; Graybiel, 2008). However, the neural correlates of encoding sequence boundaries in humans have not yet been well studied and understood. Direct local field potential recordings from the human basal ganglia—available in patients with movement disorders—demonstrated differential changes of beta (13–30 Hz) oscillatory activity for boundary and within-sequence elements (Herrojo Ruiz et al., 2014a,b). In addition, one neuroimaging study reported that during a sequential visual task, increased BOLD activity in different prefrontal areas was specifically associated with either encoding boundary elements (the mid-ventrolateral PFC) or within-sequence elements (the mid-dorsolateral PFC and anterior cingulate cortex), respectively (Amiez and Petrides, 2007). Due to their specific characteristics, namely, clinical populations and visual task, these studies provide only fragmentary evidence regarding the neural mechanisms contributing to the encoding of boundary elements during sensorimotor sequence learning in humans.

In the current study, we explored how the encoding of sequence boundaries contributes to the formation of sensorimotor sequence representations during an early phase of learning. To address this question, we recorded the neuromagnetic activity from 21 healthy subjects while they performed short piano sequences. The acquisition of representations of piano sequences relies on the encoding of the precise mapping between the motor commands for the finger movements and the monitoring of the associated auditory feedback. To investigate the encoding of boundary elements in that context, we introduced an experimental manipulation. Specifically, we examined how *alterations of feedback* (AF), introduced at different ordinal positions (boundaries [Bo] or within-sequence elements [In]), affected the neural and behavioral responses during sensorimotor sequence learning.

Notably, AF introduced at random positions during piano performance elicit a frontocentral negative-going event-related potential (ERP) peaking between 140 ms and 240 ms, termed feedback-error related negativity (fERN), and likely generated by the anterior cingulate cortex (ACC, Maidhof et al., 2010). This component is followed within 280–330 ms by a later positive deflection with fronto-central topography, the P3a, reflecting an involuntary shift in attention towards unexpected stimuli (e.g., Escera et al., 2000). The use of better spatially-resolved techniques such as fMRI to investigate AF during piano performance has confirmed the crucial involvement of the ACC in processing AF (Pfordresher et al., 2014). Additionally, AF during music performance, as well as during singing and speech induced enhanced activation in the superior temporal lobe (Tourville et al., 2008; Zarate and Zatorre, 2008; Chang et al., 2013; Pfordresher et al., 2014; Herrojo Ruiz et al., 2017).

Here, in order to demonstrate a pivotal role of boundary elements encoding in sequence learning in humans, we tested the hypothesis that feedback alterations on boundary elements would disrupt behavioral performance to a greater extent than alterations on within-sequence elements. Parallel to the changes in performance, we anticipated that the brain responses during the early acquisition of auditory-motor representations would reflect different neural processing of the AF when introduced at the start/end or within-sequence elements. Based on the reviewed literature, we hypothesized that at the neural level, changes in processing AF at the different classes of ordinal positions (boundary, within-sequence elements) would be localized in areas of the prefrontal and temporal cortices, pre- and postcentral gyri (sensory and motor areas), and the SMA. Generic processing of AF, regardless of the ordinal position, should elicit a fERN (Maidhof et al., 2010), signaling the processing of error feedback that does not match the expected feedback based on motor prediction, itself related to prediction error (Chase et al., 2011). Initial processing of the error feedback may be followed by a deflection around 300 ms, similar to the P3a, related to the automatic shift of attention to deviant stimuli (Comerchero and Polich, 1999; Maidhof et al., 2010). Thus, a combined effect on behavioral and neuromagnetic responses would underscore the prominent role of boundaries during sensorimotor sequence learning.

## Materials and Methods

### Participants

Participants in the study included 21 healthy, right-handed subjects (10 females, aged 22–34 years, mean age = 27 years) with no extensive formal piano training (accumulated lifetime practice experience below 500 h). The participants had no history of neurological or psychiatric disorders and were compensated for their participation. They all gave written informed consent, and the study was approved by the ethics committee of the University of Leipzig. The data from one participant were discarded due to bad quality of the MEG recordings. In this study, we focused on evoked magnetic fields while our previous study used the data for the analysis of neuronal oscillations (Herrojo Ruiz et al., 2017).

### Material and Procedure

**Figure 1:**
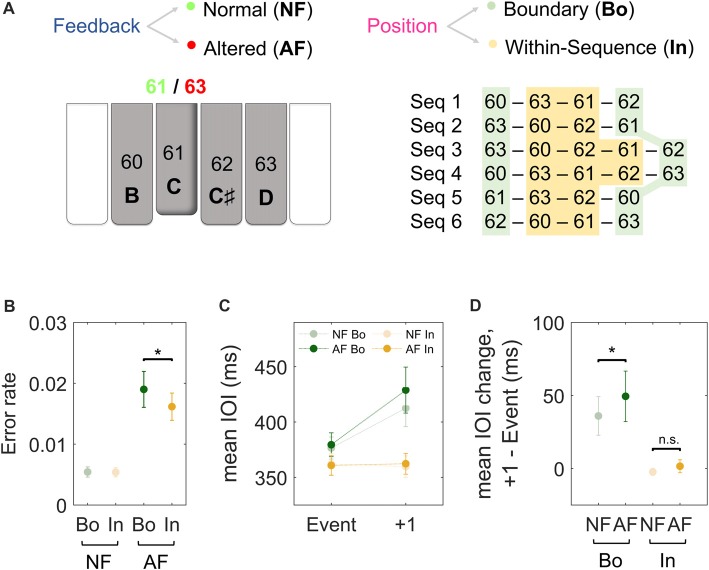
Experimental paradigm and behavioral results. **(A)** Subjects performed piano sequences 1–6 with the dominant hand while listening to the associated normal feedback (NF) and occasionally to altered feedback (AF). The pitch content (and corresponding MIDI note numbers) of our custom-made MEG-compatible keyboard is displayed at the bottom of the schematic piano keys. For instance, as illustrated in the figure, if the key 61 [C] was played, participants could listen either to the NF (61, [C]) or to some AF (e.g., 63, [D]). The AF was presented either at the Boundary (Bo, green) or Within-sequence (In, orange) elements of the sequences. Each type of sequence was performed repeatedly in a block of 15 trials of 23 s duration each. The paradigm corresponds to the one used in Herrojo Ruiz et al. (2017). **(B)** The error rates after NF (light) and AF (dark) on Bo (green) and on In events (orange). The error rate was calculated as the ratio of the number of NF and AF events that induced an error in the next five key presses over the total number of the NF and AF events, respectively.** (C)** The timing performance (mean inter-onset interval or IOI, ms) of Bo (green) or In (orange) events with NF (light) or AF (dark) and the subsequent keystroke (+1). **(D)** The figure depicts the average difference in IOI (ms) between the analyzed events and the first subsequent keystroke for all the experimental conditions. Error bars denote the standard error of the mean; *p < 0.05 (non-parametric permutation test); n.s., not significant.

This study is a re-analysis of Herrojo Ruiz et al. (2017) and the paradigm corresponds to the one used in that study, where more details are provided. The participants were asked to perform six sensorimotor sequences on a MIDI piano, using the dominant hand. The sequence patterns had a length of 4–5 notes and were explicitly taught to the participants (Figure 1A). The patterns were constructed to enable varying combinations of the successive finger movements. The keyboard did not have any ferromagnetic component and was tested for MEG and MRI compliance (Bangert et al., 2006). The time delay between keystrokes when registered as MIDI data and the corresponding trigger in the MEG recording was in the range of 20–25 ms. This interval was corrected for the MEG analyses. Accordingly, event-related field (ERF) waveforms at time 0 ms correspond with the keystroke.

There were two sessions: *familiarization* and *training*. Both these sessions corresponded to an early stage of motor skill learning, which is characterized by rapid improvements in performance (i.e., improved timing and reduced error rates; Dayan and Cohen, 2011).

In both sessions, participants played the sequences and listened to the corresponding auditory feedback, which was delivered through air-conducting plastic ear tubes. During *familiarization*, participants practiced each sequence type in a block of three trials of 23 s duration. The performance tempo was paced by a metronome on a 200 bpm (beats per minute) speed prior to each trial (see next section).

In the *training* session participants completed, for each sequence type, a block consisting of 15 trials. In each trial of duration 23 s, participants had to play that sequence type continuously without breaks. As in the familiarization session, the tempo was induced at 200 bpm with the use of a metronome prior to each trial. Participants were instructed to play continuously during the trial’s length without stopping to correct any errors.

During the task, in 12 out of 15 trials for each sequence type, alterations of auditory feedback (termed, alterations of feedback, AF) were introduced randomly between every 8th and 10th produced note (every 8.37 [standard error of the mean or SEM, 0.05] keystrokes on average). We used this design because lower AF rates do not lead to behavioral effects (Maidhof et al., 2010; Pfordresher and Kulpa, 2011). The AF coincided with either boundary (*Bo*) or within-sequence (*In*) elements of the sequence. In the events with modified feedback, instead of hearing a tone corresponding to a given pressed key, participants heard either the tone of a different element of the sequence being played (i.e., from a different serial position in the sequence) or a tone that was unrelated to the sequence content. For a differential analysis of the two types of perturbations, see Herrojo Ruiz et al. (2017). Participants were informed, prior to the task, about the occasional occurrence of feedback alterations. Trials 1, 6 and 10 were perturbation-free.

### Behavioral Analysis

General performance was evaluated by measuring three parameters, the average timing (time between consecutive keystrokes or inter-onset interval, IOI, in ms), the variability of timing (coefficient of variation for IOI [cvIOI]), and the error rate.

Previous studies focusing on sensorimotor sequence learning during an early training phase support the dissociation of two processes: (a) the encoding of the serial order of the actions (spatial feature), more strictly related to learning and reflected in error rates; (b) the concurrent improvements in performance, as reflected in faster tempo or reaction times and reduced temporal variability with training (Seidler et al., 2002; Kornysheva and Diedrichsen, 2014). Accordingly, to test the effects of AF on sequence learning, we used as main dependent variable the rate of pitch errors induced in the subsequent key presses. Specifically, the error rate was calculated as the ratio of the number of AF events that induced an error in the next five key presses over the total number of AF events. AF events that were followed by another AF event in the considered range—five subsequent keystrokes—were excluded from the error rate analysis, as the error could have been induced by the subsequent AF event. In addition, the effects of feedback alterations on the performance changes that typically accompany sequence learning (i.e., encoding) were assessed in terms average tempo and cvIOI in AF trials, as well as post-feedback slowing (larger IOI in events following the AF event).

### MEG Data Acquisition and Pre-processing

Neuromagnetic signals were recorded during the performance session using a 306 sensor Elekta Neuromag system (Elekta Neuromag Oy, Helsinki, Finland) in an electromagnetically shielded room (Vacuumschmelze, Hanau, Germany). The MEG device has 102 triple sensor elements in a head-shaped array, and each of the elements was comprised of one magnetometer and two orthogonal planar gradiometers.

Head Position Indicator (HPI) coils attached to the scalp were used to monitor head movements. Vertical and horizontal bipolar electrooculograms (EOG) and electrocardiograms (ECG) were recorded simultaneously with the MEG recording so that we could control for ocular and cardiac artifacts.

Magnetic signals were recorded at a 1000 Hz sampling rate and a low pass filter of 330 Hz. The signal space separation method (Maxfilter Neuromag; Taulu et al., 2004) was used to suppress extracranial noise and to project individual signal space data to a default head position. This allowed performing statistical analyses across participants in sensor space. An additional correction was applied to one participant (#15) whose head displacement was larger than 5 mm (temporal-spatial filtering algorithm, MC Neuromag, Taulu and Kajola, 2005; Taulu and Simola, 2006). On average, the head displacement in all participants was 1.8 (standard error of the mean or SEM, 2) mm (range 0.5–4 mm; excluding Participant 15).

Further analysis was performed with custom-made Matlab algorithms (The MathWorks Inc., MA, USA) and the Fieldtrip toolbox (Oostenveld et al., 2011). The analysis was restricted to the 204 planar gradiometer sensors because they are more sensitive to cortical sources directly underneath them and less sensitive to extracranial noise sources (Hämäläinen et al., 1993). The continuous MEG data were filtered with a high pass filter of 1 Hz and a low pass filter of 100 Hz to minimize high frequency noise from MEG coils (Linear-phase FIR [Finite Impulse Response] filter as implemented by Fieldtrip with “firls” option, filter order = 6). Ocular and cardiac artifacts were identified and removed using the independent component analysis (FastICA, symmetric approach, with the hyperbolic tangent—*tanh*—as nonlinear function; Hyvärinen and Oja, 2000).

### Sensor Space: Event-Related Fields

To analyze the ERFs in the sensor space, we segmented continuous data into epochs from −1 s to 1 s, time-locked to correctly played keystroke events in the different experimental conditions. The four experimental conditions included (Figure 1A): (i) boundary events with normal feedback (NF Bo); (ii) within-sequence events with NF (NF In), (iii) boundary events with AF (AF Bo); and (iv) within-sequence events with AF (AF In). For the conditions with AF, AF Bo and AF In, approximately 135 and 170 artifact-free epochs were extracted on average, respectively. From the larger pool of events (>1000) corresponding to the conditions with NF (NF Bo, NF In), we extracted the same number of events as those available in the AF conditions (135 and 170 on average, respectively). NF events following AF events (+1, +2) were excluded from this selection process. Importantly, the selected epochs were matched in timing (IOI) and keystroke velocity to the epochs from the AF conditions. These selected epochs were visually inspected for further artifacts. After visual inspection, 123 (SEM, 4) trials on average remained for NF Bo, 123 (4) for AF Bo and 157 (6) and 156 (6) for NF In and AF In, respectively. Finally, the average ERF across trials was estimated relative to a pre-keystroke baseline (−200 to −100 ms) and separately for each condition and participant. The data from the planar gradiometers were then combined at each sensor position by computing the mean square root of the signals (“combined planar” representation in FieldTrip).

### Source Reconstruction

To examine between-condition differences at the source level, we calculated the current distribution of the sources with the use of L2-norm minimum-norm estimates (MNEs, Hämäläinen and Ilmoniemi, 1994; Dale et al., 2000). Note that MRI segmentation, coregistration and forward model estimation were performed as in Herrojo Ruiz et al. (2017). In brief, we first processed the individual T1-weighted MRI images (3T Magnetom Trio, Siemens AG, Germany) with the “Freesurfer” software^1^ for segmentation of the MRI data and cortical surface reconstruction. The “MNE” software^2^ was then used for the co-registration of the MR and MEG coordinate systems and the construction of boundary element conductivity models (BEM) to use in the forward calculations. We selected the inner skull surface as volume conductor geometry. Then we created a cortical grid in the MNI space template brain (as used in SPM8) with 4 mm resolution as the respective source space. We further warped this grid into the subject-specific space by use of transformation matrices obtained during the normalization of individual MR images. The source space, the volume conductor model, and the position of the planar gradiometer sensors were then used for the calculation of the forward model. In the last step, we computed the inverse solution using the L2-norm MNE method, as implemented in the FieldTrip software (minimum-norm estimate, based on Dale et al., 2000; Lin et al., 2004). MNE sources were estimated for each grid point at the time interval between 0.15 s and 0.37 s post-keystroke, and the individual source solutions were interpolated to a template MNI mesh. The noise-covariance matrix was estimated for each subject using data from the time intervals preceding each performance trial (thus corresponding to periods of no performance, amounting to ~3–5 min). The noise-covariance matrix was scaled using the regularization parameter λ (as in Dale et al., 2000; Lin et al., 2004). Here λ was set to the value recommended in the FieldTrip tutorial (initially, though, we explored different values of λ, which did not change the results qualitatively).

### Statistical Analysis

To test for main effects and interactions of factors Feedback (normal, altered) and Position (boundary, within-sequence) in the behavioral data, we first run a 2 × 2 non-parametric factorial analysis (synchronized rearrangements, Good, 2005; Basso et al., 2007). This analysis was complemented with *post hoc* pair-wise permutation tests across subjects (Good, 2005) to assess significant differences between experimental conditions (e.g., error rates or mean IOI following AF at Bo and In). The difference in sample means was the test statistic. We performed *n* = 5000 rearrangements, drawn at random from the complete permutation distribution (Monte Carlo permutation test). The *p-values* were estimated as the percentage of the replications of the test statistic that had absolute values larger than the experimental difference.

To assess differences in the ERFs at the sensor level, we performed a 2 × 2 non-parametric factorial analysis with factors Feedback and Position. This analysis focused on the time interval from 0.15 s to 0.37 s post-keystroke. The choice of this time interval was primarily based on previous research suggesting that the main effects associated with the processing of AF during performance occur approximately between 0.15s and 0.25 s after the AF (Maidhof et al., 2010). However, we extended the window of analysis to 0.37 s because this was the average IOI during performance. Accordingly, the choice of this upper limit enabled the investigation of ERF effects up to the next keystroke.

To correct for multiple comparisons, we controlled the false discovery rate (FDR) at level *q* = 0.05 by using an adaptive two-stage linear step-up procedure (Benjamini et al., 2006). The result of this procedure is the corrected threshold *p*-value, which is provided in the text as *pthr*, when multiple comparisons were performed. Note that as a sanity check we also ran statistical tests in the peri-keystroke interval [−200, +150 ms] and found no significant clusters (*p* > 0.05).

*Post hoc* analyses of the ERFs following the 2 × 2 factorial analysis were performed in the same time window 0.15–0.37 s with non-parametric tests based on spatio-temporal clustering, using the FieldTrip software (dependent samples *t*-test, 1000 iterations; Maris and Oostenveld, 2007). The threshold to control for family-wise error (FWE) was set to *p* = 0.025 (two-sided test). The test statistic of the observed data was evaluated against the Monte-Carlo permutation distribution in order to test the null hypothesis of no difference between conditions. We applied the cluster-based permutation tests to the following between-condition differences: (i) AF Bo − NF Bo; (ii) AF In − NF In, to test for Feedback effects (Altered − Normal); (iii) NF Bo − NF In; and (iv) AF Bo − AF In to test for differences related to the ordinal Position (boundary − within-sequence).

Following up on the finding of a significant interaction between factors Position and Feedback in the sensor space (see “Results” section), statistical analyses at the source level focused on the pair-wise comparisons outlined in the previous paragraph for the ERF analysis and were carried out with pair-wise permutation tests.

To estimate statistically dynamic changes at the source level, we divided the time interval 0.15–0.37 s into four segments of 55 ms-length each and calculated the average source current distribution for each segment. We then applied the permutation test to assess between-condition source differences in each segment separately. The same design as the one used in the cluster statistics (described in the previous paragraph) was used to examine potential effects on the source level related to AF and ordinal position. The test was repeated for each grid point of the cortical mesh (~8000), and to correct for multiple comparisons, we kept the FDR at level *q* = 0.05, as indicated above. The anatomical locations with significant differences in source activity were identified by the use of the Automated Anatomical Labeling atlas (AAL). The regions of interest included the pre- and postcentral gyri, corresponding to sensory and motor areas, and the frontal and temporal cortices. Furthermore, the “area of significant activation” was estimated by the ratio of the number of significant grid points over the total number of grid points for each region (in percentage). In Tables 1, 2 we report the cortical regions with an “area of significant activation” equal or larger to 10%.

**Table 1:** Feedback, altered feedback (AF) vs. normal feedback (NF).

Side	Region	MNI coordinates (mm)			Activation strength (nAm)	Time segments
		*x*	*y*	*z*		
**Boundaries**						
**AF > NF**						
R	Postcentral	45	−23	38	1.92	S3, S4
R	Temporal Sup	53	−19	2	2.44	S2, S3, S4
R	Frontal Inf Oper	42	11	26	0.85	S3, S4
R	Temporal Inf	52	−21	−27	1.31	S3, S4
R	Temporal Mid	55	−19	−13	2.09	S3, S4
R	Precentral	46	−8	33	1.55	S4
**Within-Sequence**						
**AF > NF**						
R	Temporal Sup	55	−23	5	3.55	S3, S4
R	Postcentral	51	−18	37	3.60	S3, S4
L	Temporal Sup	−52	−24	7	2.29	S3, S4
L	Temporal Mid	−54	−31	−2	2.60	S2, S3, S4
L	Frontal Inf Tri	−45	25	11	1.42	S3, S4
R	Precentral	48	−6	40	3.33	S3, S4
L	Temporal Inf	−42	−5	−37	0.80	S3, S4
R	Frontal Inf Oper	47	12	19	2.17	S3, S4
R	Frontal Inf Tri	48	24	18	3.03	S4
L	Frontal Inf Orb	−36	25	−13	0.99	S3, S4
R	Temporal Mid	56	−39	1	2.48	S1, S2, S3, S4
L	Frontal Inf Oper	−45	12	18	1.56	S3, S4
L	Temporal Pole Sup	−40	10	−21	1.02	S3, S4
L	Postcentral	−57	−13	19	3.70	S3, S4
L	Cingulum Mid	−8	−6	40	0.16	S3, S4
L	Precentral	−48	2	27	1.89	S4
L	Temporal Pole Mid	−36	9	−35	0.76	S3, S4

*Brain regions showing significant differential activation between Bo and In elements. Coordinates represent the average across all significant grid points for each region. “Activation Strength” is the average source activation across all the region-specific significant grid points. “Time segments” indicate the time windows in which the reported regions yielded significant effects. S1, 150–205 ms; S2, 205–260 ms; S3, 260–315 ms; S4, 315–370 ms; L, Left; R, Right*.

**Table 2:** Position, Boundary (Bo) vs. Within-Sequence (In).

Side	Region	MNI coordinates (mm)			Activation strength (nAm)	Time segments
		*x*	*y*	*z*		
**Normal feedback**						
R	Frontal Mid	35	30	29	1.65	S1(+)
L	Temporal Mid	−54	−21	−12	1.30	S1(+)
R	Frontal Sup	22	28	37	1.63	S1(+)
L	Temporal Inf	−45	−14	−31	0.59	S1(+)
R	Precentral	43	−7	45	1.44	S1(+)
L	Cingulum Ant	−7	27	16	0.17	S1(+)
L	Precentral	−36	−9	46	1.77	S1(+)
R	Temporal Mid	46	−52	6	0.74	S1(+), S2 (+)
L	Frontal Mid	−29	37	24	1.46	S1(+)
R	Cingulum Mid	8	−2	35	0.18	S1(+)
L	Temporal Sup	−53	−28	6	1.54	S1(+)
R	Cingulum Ant	8	31	14	0.20	S1(+), S3 (−)
L	Frontal Inf Tri	−44	24	17	1.76	S1(+)
L	Frontal Sup Medial	−8	49	21	1.00	S1(+)
R	Frontal Inf Orb	35	29	−14	0.50	S1(+), S3 (−)
R	Frontal Inf Tri	41	28	17	1.20	S1(+)
R	Frontal Sup Medial	10	55	18	1.10	S1(+)
L	Frontal Sup	−20	36	33	1.93	S1(+)
L	Frontal Inf Oper	−47	11	19	2.20	S1(+)
R	Frontal Mid Orb	31	49	−11	0.56	S1(+)
R	Supp Motor Area	10	−4	57	1.64	S1(+)
R	Frontal Sup Orb	19	43	−16	0.23	S1(+), S3 (−)
L	Cingulum Mid	−8	−3	36	0.17	S1(+)
L	Temporal Pole Sup	−40	7	−23	0.34	S1(+)
R	Temporal Inf	46	−5	−37	0.93	S1(+)
R	Postcentral	55	−12	35	1.56	S1(+)
L	Frontal Inf Orb	−31	28	−16	0.38	S1(+)
R	Temporal Pole Mid	39	9	−35	0.57	S1(+)
L	Frontal Sup Orb	−20	47	−11	0.41	S1(+)
L	Frontal Mid Orb	−29	47	−11	0.50
**Altered feedback**						
L	Temporal Mid	−53	−26	−7	−0.03	S1(+), S3 (−)
L	Temporal Sup	−52	−21	3	−0.81	S1(+), S3 (−)
L	Frontal Mid	−32	29	33	2.36	S1(+), S3 (−)
L	Temporal Inf	−48	−16	−29	0.31	S1(+), S3 (−)
R	Frontal Sup	22	34	31	1.37	S1(+), S3 (−)
R	Frontal Mid	34	33	28	1.48	S1(+)
L	Temporal Sup	−53	−28	6	1.54	S1(+)
L	Cingulum Ant	−7	30	17	0.15	S1(+), S3 (−)
L	Postcentral	−52	−18	28	−0.69	S1(+), S2(+), S3(−), S4(−)
L	Frontal Inf Tri	−43	24	18	1.63	S1(+), S3 (−)
R	Frontal Inf Orb	37	30	−13	0.04	S1(+), S3 (−)
L	Frontal Inf Oper	−46	12	19	0.74	S1(+), S3 (−)
L	Frontal Sup	−18	42	33	2.28	S1(+), S3 (−)
L	Frontal Sup Medial	−8	50	24	1.38	S1(+)
R	Frontal Sup Medial	10	56	14	0.96	S1(+), S3 (−)
R	Precentral	42	−9	48	0.68	S1(+), S3 (−)
R	Cingulum Ant	9	38	11	0.21	S1(+), S3 (−)
L	Temporal Pole Sup	−40	7	−23	0.34	S1 (+)
R	Supp Motor Area	10	1	59	−1.08	S1(+), S3 (−)
R	Frontal Mid Orb	32	50	−11	0.51	S1(+), S3 (−)
R	Temporal Pole Mid	39	10	−34	−0.24	S1(+), S3 (−)
R	Frontal Sup Orb	19	42	−16	0.19	S1(+), S3 (−)
R	Temporal Pole Sup	40	9	−24	0.06	S1(+), S3 (−)
L	Precentral	−47	3	27	0.64	S1(+), S3 (−)
R	Temporal Inf	43	0	−40	−0.63	S1(+), S3 (−)

*Brain regions showing significant differential activation between Bo and In elements. Coordinates represent the average across all significant grid points for each region. “Activation Strength” is the average source activation across all the region-specific significant grid points. “Time segments” indicate the time windows in which the reported regions yielded significant effects. The sign in the parentheses indicates the direction of the effect in the particular time segment (“+” for Bo > In and “−” for Bo < In). S1, 150–205 ms; S2, 205–260 ms; S3, 260–315 ms; S4, 315–370 ms; L, Left; R, Right*.

In addition to significance testing using permutation tests, a nonparametric effect size estimator, the probability of superiority for dependent samples or PS_dep_ (Grissom and Kim, 2012), is reported. PS_dep_ is an estimation of the probability (maximum 1) that in a randomly sampled pair of matched values (from same individual), the value from Condition B will be greater than the value from Condition A: PS_dep_ = Pr (XiB > XiA). Throughout the manuscript, this index will be provided in association with each pair-wise permutation test.

## Results

### Behavioral Results

Data are provided as the mean and, in parentheses, SEM. Figure 1C depicts the average timing (IOI) of the analyzed events and the subsequent keystroke for all conditions, while Figure 1D depicts the average IOI difference between the two. We first compared the timing performance (mean IOI, ms) between events with NF and AF and, expectedly, found no significant differences (*p* > 0.05 overall, but also for all positions of 4-note sequences and 5-note sequences). To investigate the effect of AF on the timing of the subsequent keystrokes, and the role of ordinal position, we analyzed the change in the mean IOI (ms) at the keystroke following the feedback. A 2 × 2 factorial analysis of the timing performance at the subsequent (+1) stroke with factors Feedback (NF, AF) and Position (Bo, In) demonstrated a significant main effect for both factors (Feedback, *p* = 0.0064; Position, *p* < 0.001) as well as an interaction effect (*p* = 0.008). With respect to the main effect of Position, *post hoc* comparisons revealed that IOI change at +1 keystroke was significantly larger for Bo compared to In, both when AF and NF were introduced (AF: 49 [16] ms for Bo and −2.1 [3.7] ms for In, *p* < 0.001, PS_dep_ = 0.85; NF: 34 [16] ms for Bo and −3.8 [2.4] ms for In, *p* < 0.001, PS_dep_ = 0.95). Regarding the main effect in Feedback, *post hoc* comparisons were performed for IOI changes at +1 (Bo: AF vs. NF and In: AF vs. NF) in order to investigate whether the manifestation of AF-induced changes in tempo were different depending on the position in the sequence on which AF was introduced (Bo or In elements). When introduced at Bo elements, AF caused a slowing at the subsequent keystroke (an IOI difference of 49 [16] ms), which was significantly larger than the change in IOI observed at the same position following NF on Bo elements (34 [16] ms; *p* = 0.009, PS_dep_ = 0.65; Figure 1D. Note that any changes in IOI following boundary elements with NF are due to sequence-position-specific characteristics). A similar analysis performed for In elements under AF showed that the effect of AF on the timing (IOI) of the subsequent keystroke was not significantly different relative to the timing (IOI) change at the same position following NF on In elements (*p* > 0.05; Figure 1D). The comparison of the difference (AF-NF) between Bo and In did not reach significance level (15 [6.2] ms for Bo and 1.7 [2.7] for In, *p* = 0.08, PS_dep_ = 0.65). A detailed separate analysis for each ordinal position (Bo: first and last elements; In: each element within the sequence) confirmed this pattern of results and can be found in Supplementary Figure S1. In summary, these results show that the timing performance at the first subsequent keystroke is disturbed only after introducing AF on Bo but not on In elements.

With regard to the error rates, a 2 × 2 analysis on the error rates following Bo and In elements (for AF and NF events) unveiled a significant main effect for factor Feedback (*p* = 0.009) and a significant interaction (*p* = 0.013). This main effect was due to larger error rates following AF events (0.011 [0.02] on average) than NF events (0.005 [0.002]). When exploring the interaction effect, we found that AF induced a larger error rate when introduced at Bo elements relative to In elements (0.019 [0.003] for Bo and 0.016 [0.002] for In; *p* = 0.04, PS_dep_ = 0.74; Figure 1B).

In sum, our findings on timing and error rates suggest that behavioral changes due to AF are sensitive to the class of ordinal position and that feedback modifications affecting start and end (boundary) elements seem to disrupt behavior to a greater extent than when introduced at within-sequence elements.

Lastly, we assessed sequence-specific learning and related changes in performance across time in purely NF trials, which are better suited to test effects of training when AF is not present (see also Herrojo Ruiz et al., 2017, for more details). There was no significant change in error rates from the first (#1) to the last (#11) NF trial (*p* > 0.05). Regarding changes in timing in NF trials across time, we found no significant changes in tempo but a significant increase in the extent of temporal variability (cvIOI = 0.24 [0.02] in trial 1 and 0.31 [0.03] in trial 11, *p < pthr* = 0.0028, PS_dep_ = 0.80).

A similar analysis performed across time throughout the experiment, regardless of the sequence that participants played (i.e., comparison of data in the first and second half of the experiment), revealed also no significant changes in error rates in NF trials (*p* > 0.05). Notably, however, across time throughout the experiment there was a significant improvement in the average timing performance (mean IOI from 395 [10] ms to 374 [9] ms; *p* = 0.001, PS_dep_ = 0.90).

### Sensor Space: Event-Related Fields

**Figure 2:**
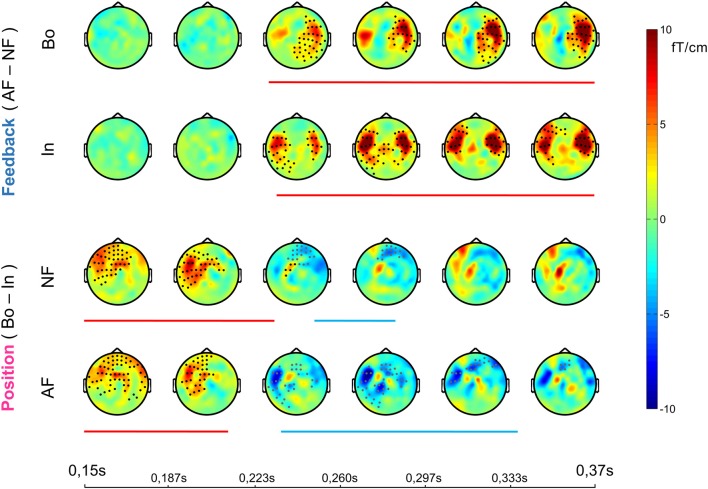
Sensor-level event-related field (ERF) effects. Each row shows the temporal evolution of the topography of the average ERF differences between the compared conditions in the window 0.15–0.37 ms post-keystroke. Cluster statistics were performed on that time window to explore possible significant effects for all of the four *post hoc* comparisons (1st row, AF Bo − NF Bo; 2nd AF In − NF In; 3rd NF Bo − NF In; 4th AF Bo − AF In). The lines below each row show the duration of each significant cluster (red denotes a positive and blue a negative effect) and the dots on the topography maps indicate the sensors that contribute to the cluster (black for positive and gray for negative cluster).

**Figure 3:**
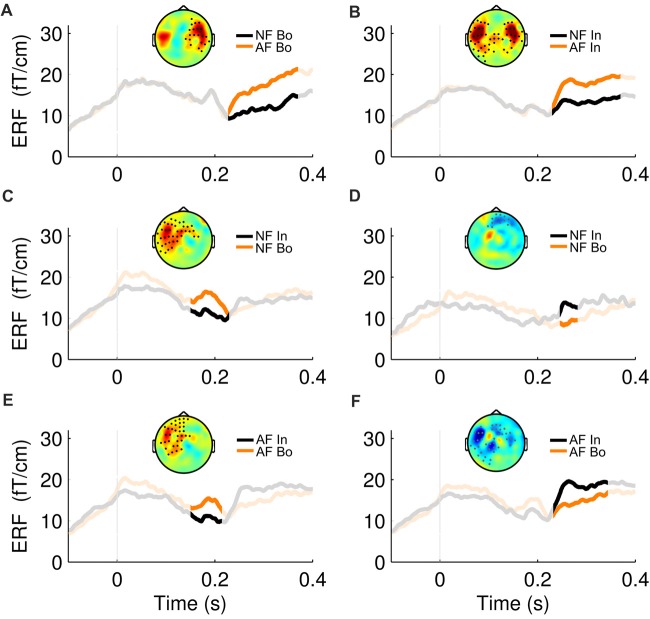
ERF waveforms for the significant effects. For all the significant effects revealed by the cluster statistical analysis (2 for Feedback and 4 for Position comparisons, see Figure 2), an exemplary topographical map of the ERF difference between the two compared conditions as well as their ERF waveform, averaged over the significant sensors, is shown here. The non-shaded area in each plot represents the duration of the respective cluster. The first row represents the significant effects in the “Bo: AF—NF” **(A)** and “In: AF—NF” **(B)** comparisons. The second and third row correspond to the positive (left) and negative (right) effects found in the “NF: Bo—In” **(C,D)** and “AF: Bo—In” **(E,F)** comparisons, respectively.

The effects of AF and the ordinal position on the averaged ERFs were examined using a 2 × 2 non-parametric permutation-based factorial analysis with factors Feedback (normal, altered) and ordinal Position (Bo, In). This test revealed a significant main effect for both factors and a significant interaction within 150–370 ms post-keystroke (*p < pthr* = 0.0032 for interaction, 0.0050 for factor feedback and 0.0016 for factor position). *Post hoc* analyses with cluster-based permutation tests—performed in the same time window (150–370 ms)—revealed for both Feedback comparisons AF Bo − NF Bo and AF In − NF In a significantly larger activation for the AF conditions (*p* < 0.025). The significant difference was mainly due to enhanced activation over right (AF Bo − NF Bo) and bilateral (AF In − NF In) temporal sensors (Figure 2, Feedback, Figures 3A,B). A further comparison of the AF-NF difference between Bo and In (double difference statistic) was not significant. The cluster analysis for the comparisons NF Bo − NF In and AF Bo − AF In, which was performed to investigate the effect of ordinal position, demonstrated for both comparisons one positive and one negative significant cluster (*p* < 0.025; positive means Bo > In and negative Bo < In). In both cases, the differences were more pronounced over fronto-temporal and central sensors (Figure 2, Position, Figures 3C–F). Overall, this analysis revealed at the sensor level a differential activation pattern for the contrasted conditions in both the examined factors Feedback (AF vs. NF) and ordinal Position (Bo vs. In).

### Source Space Analysis

**Figure 4:**
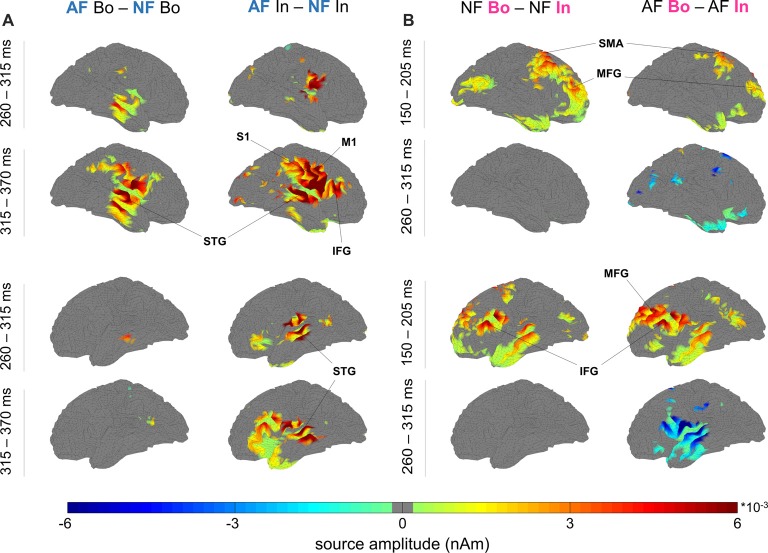
Neural sources of the differential response to AF vs. NF and boundary vs. within-sequence items. **(A)** Feedback: significant source activity differences for “AF—NF”, separately for boundary (Bo, left) and within-sequence (In, right) elements. The maps depict the cortical sources for the right (top four maps) and left hemisphere (bottom four maps) in the windows 260–315 ms and 315–370 ms. **(B)** Ordinal Position: significant source activity differences for “Bo—In” (left NF, right AF) occurred at 150–205 ms (Bo > In) and 260–315 ms (Bo < In; criterion for visualization in both figures: *p < pthr*, non-parametric permutation test, *pthr* estimated to correct for multiple comparisons, see “Materials and Methods” section).

Using minimum-norm estimates, we then contrasted the experimental conditions to define potential differences at the source level and their spatio-temporal characteristics. The selected time window from 0.15 s to 0.37 s was segmented into four epochs (S1–S4, see “Materials and Methods” section), and the source analysis results for each segment were contrasted between the compared conditions using a non-parametric permutation test. In Table 1 (Feedback effects) and Table 2 (Position effects), we report the cortical regions that showed significantly different activity between the contrasted conditions. The last column of the Tables indicates the time segments in which a particular region showed a significant effect while the sign in parenthesis in Table 2 (Position effects) indicates the direction of the effect. In the cases where an area is marked with “S1(+), S3 (–)”, that means that the particular region showed a Bo > In significant activation in the first segment (S1) while in segment S3, it showed an opposite activation pattern (Bo < In). Figure 4A illustrates the significant brain activations in the contrast between AF and NF (AF Bo − NF Bo, left panel; AF In − NF In, right panel). Table 1 indicates all the activated regions (*p < pthr*, see Table 3 and area of significant activation larger than 10% of the region’s total number of grid points). Both Feedback contrasts showed differential activation in a number of cortical locations primarily in the last two segments (S3: 260–315 ms, Figure 4A, first and third row for right and left hemisphere, respectively; S4: 315–370 ms, Figure 4A second and fourth rows), being consistent with our findings at the sensor-level (see Figure 2, Feedback). The main brain areas contributing to the significant AF minus NF contrasts were the primary sensorimotor (pre- and postcentral gyri), temporal (e.g., superior temporal gyrus) and frontal (mainly portions of the inferior frontal gyrus [IFG]) cortices. This result reflects a complex activation pattern involved in the processing of AF, which was most prominently right-lateralized for the Bo: AF-NF contrast (bilateral for the In contrast). In the right hemisphere, there was a large overlap in the implicated regions between the AF-NF contrasts for Bo and In elements. An additional comparison of the difference AF-NF between Bo and In revealed no significant effects.

**Table 3:** Corrected *p*-threshold values for source analysis.

	Segment			
	S1 150–205 ms	S2 205–260 ms	S3 260–315 ms	S4 315–370 ms
Comparison				
**Feedback**				
AF Bo—NF Bo	0	0	0.0018	0.0070
AF In—NF In	0	0	0.0030	0.0136
**Position**				
NF Bo—NF In	0.0200	0	0	0
AF Bo—AF In	0.0094	0	0.0064	0

The analysis of the factor Position revealed a significant Bo—In effect mainly localized to bilateral fronto-temporal areas, including temporal regions and the IFG—similar to the analysis of factor Feedback—but also extending substantially to the dorsolateral and medial prefrontal cortex (DLPFC, MPFC; Figure 4B, Table 2). In the first segment (S1: 150–205 ms, Figure 4B, Table 2), both for AF and NF Bo—In contrasts, frontal regions such as the bilateral IFG, DLPFC, MPFC and the right SMA as well as several temporal areas (e.g., the left middle temporal gyrus) showed a significantly larger response at Bo relative to In elements. In contrast, in the third segment (S3: 260–315 ms, Figure 4B, second and fourth rows, Table 2) of the AF Bo—In contrast, we identified a significantly weaker response at Bo compared to In elements in temporal areas (e.g., the left superior and middle temporal gyri). Overall, the results of the source reconstruction indicate, first, that the processing of feedback alterations was associated with neural activity distributed over a wide cortical network including temporal regions and portions of the IFC. Additionally, the results show that fronto-temporal regions, similar to those found for the feedback analysis but additionally extending to the (DLPFC and MPFC), were differentially activated during the encoding of boundary elements, compared to within-sequence elements. The last finding, in addition to the results from the sensor-level and the behavioral analysis, suggests a distinct role for the encoding of boundaries during sensorimotor sequence learning in humans.

## Discussion

Does the encoding of boundary and within-sequence elements differ during sensorimotor sequence learning? To address this question, the current study compared the effects of alterations of auditory feedback when introduced at boundary and within-sequence elements during the learning of short piano sequences. In terms of behavior, we found that AF led to greater disruptive changes in performance when applied at boundary relative to within-sequence elements. At the neural level, we also found a dissociation in the neural responses in the two conditions, demonstrated by the ERF and the source reconstruction analysis. Our findings thus suggest that the encoding of boundary elements plays an essential role during the early-stage acquisition of sensorimotor sequences.

### Altered Feedback at Boundary Elements Impairs Performance to a Greater Extent

The analysis of performance revealed that AF when introduced at boundary elements—compared to within-sequence elements—resulted in larger error rates and a larger post-feedback slowing in the subsequent keystroke. This finding indicates that disturbances of the action-perception contingencies during the encoding of boundaries can affect both the accuracy and the timing of behavior. Although the slowing down in timing performance induced by AF might not be a direct indicator of impaired learning, the larger number of errors after AF at boundaries demonstrates that disturbance of the encoding of boundaries leads to greater disruption of sequential learning. This interpretation is aligned with the reported dissociation between error rates—as an index of sequence encoding and learning—and the changes in the timing of performance that occur in parallel (e.g., Seidler et al., 2002). Thus, the evidence supports that the adequate encoding of start and end elements facilitates sensorimotor sequence learning.

### Fronto-Temporal and Sensorimotor Responses to Altered Feedback (AF)

With respect to the neuromagnetic processing of AF, the present data showed that AF activated the auditory cortex, the IFG, and the pre- and post-central gyri. The temporal cortex is involved in processing musical sequences (Hickok et al., 2003; Koelsch et al., 2005) and auditory sequence violations (Giard et al., 1995; Uhrig et al., 2014). In addition, it is critical for auditory-motor transformations in speech and aspects of musical abilities (Hickok and Poeppel, 2004). Although a recent fMRI study employing a similar design (Pfordresher et al., 2014) reported Spt (Sylvian parieto-temporal area) but no temporal cortex activation in response to AF, this might be due to the low sensitivity of fMRI in detecting rapid changes in brain activity, which were, however, detectable in the current experiment. The IFG (BA44) is involved in syntax processing during music performance and perception (Maess et al., 2001; Bianco et al., 2016) while music-related auditory-motor transformations involved IFG as well as the premotor cortex (Bangert et al., 2006; Lahav et al., 2007). Significantly, an earlier model of musical processing by Koelsch (2005) suggested that musical syntax engages the inferior frontolateral cortex (BA44), the premotor cortex and STG with a right hemispheric dominance. Because our sequences did not follow any harmonic rules but were instead created as simple associations between movement and pitch values, our findings expand upon the proposal by Koelsch et al. (2005) in demonstrating the involvement of these structures in processing more generic auditory-motor associations. This interpretation thus aligns well with anatomical data from non-human primates showing that STG is directly connected to IFG and the premotor cortex (see Zatorre et al., 2007).

We additionally found simultaneous activation of pre- and post-central gyri, which have been reported in previous studies to be activated during music performance and imagery (Meister et al., 2004) and during auditory feedback control in speech (Tourville et al., 2008). Thus, this finding might reflect the activation of the sensorimotor network that is known to connect sensory and the motor cortex during motor behavior (e.g., Brovelli et al., 2004).

Another interesting finding of the source analysis was a difference in laterality for the effects of AF depending on the position on which AF was introduced. While AF on within-sequence elements resulted in the engagement of bilateral fronto-temporal areas, the processing of feedback alterations on Bo was limited to right fronto-temporal areas. This difference in laterality could reflect a more localized processing of disturbances of boundary encoding as opposed to within-sequence elements. However, clarifying the implications of this laterality effect will require follow up studies aiming to replicate our findings.

### DLPFC and SMA Support the Differential Encoding of Ordinal Position

The main aim of the study was to investigate whether different neural mechanisms are involved in the encoding of boundary and within-sequence elements and their disruption by AF. We found evidence supporting differences in the event-related responses to boundaries and within-sequence elements under AF as demonstrated by an early fronto-temporal positivity and a subsequent negativity over mainly temporal sensors. The source reconstruction analysis suggested that the latter effect emerges around 260–315 ms and stems mainly from the left temporal cortex. Although a similar negativity in the ERF was observed also for the NF at the sensor level, the effect was shorter in time and spatially more confined. Note, however, that this effect had no corresponding significant source. This negative ERF pattern found in the Bo—In contrast might be associated with the interaction effect as it extended over a longer period and was more pronounced in AF than NF conditions. Note, however, that our *post hoc* exploration of the interaction effect (factors Feedback—AF, NF—and Position—Bo, In) did not reveal conclusive results concerning the direction of changes leading to the interaction effects.

In contrast, the early fronto-temporal positivity had the same spatio-temporal characteristics under both NF and AF, suggesting that it might index merely different encoding of boundary and within-sequence elements regardless of the auditory feedback. The source analysis supports this idea by revealing a large overlap of activated regions in the corresponding time window between the NF Bo—NF In and AF Bo—AF In contrasts. This activation was distributed predominantly over the bilateral DLPFC, an area which has been consistently implicated in different aspects of serial order encoding both in human and non-human primate studies (Averbeck et al., 2002; Ninokura et al., 2004; Fujii and Graybiel, 2005; Amiez and Petrides, 2007). The observed additional activation of the right SMA is in agreement with studies linking SMA to sequential behavior (Shima and Tanji, 2006) and proposing a key role for SMA in the encoding of temporal structure (Kotz and Schwartze, 2011). The left IFG and temporal regions that were also activated have been related to auditory chunking (Dehaene et al., 2015) and formation of structural representations (only left IFG; Karuza et al., 2013), and thus they probably act complementarily to DLPFC and SMA. The present findings provide a clear indication for the differential encoding of boundaries and within-sequence elements during the early stage of sensorimotor sequence learning, a process that is supported by the DLPFC and SMA with the IFG and temporal regions having a complementary role.

Previous studies have demonstrated that the basal ganglia are involved in the encoding of sequence boundaries during the acquisition of sequential behavior (Jin and Costa, 2010; Herrojo Ruiz et al., 2014a,b). This finding converges with similar evidence for the frontal (Fujii and Graybiel, 2005) and motor cortex (Santos et al., 2015). We therefore propose that DLPFC and SMA might interact with the basal ganglia via cortico-basal ganglia loops during the differential encoding of boundary and within-sequence elements during the early phase of sequence acquisition. Ultimately, this encoding leads to the concatenation of actions into integrated units of behavior (Graybiel, 2008).

### Methodological Considerations

As in any MEG study, the results of the source localization should be interpreted with caution, given the limitations that affect source localization of MEG data (Hari et al., 1988; Hansen et al., 2010). Moreover, it should be noted that minimum-norm estimates are limited to the cortical surface, thereby excluding the possibility of detecting other structures relevant for processing feedback-related errors in our paradigm, such as the cerebellum (Herrojo Ruiz et al., 2017). In addition, a higher sensitivity to neuromagnetic sources in the cerebellum might be better achieved by focusing on high-frequency oscillatory activity (E/MEG: Dalal et al., 2008, 2013).

Interestingly, a few recent studies have reported the involvement of the cingulate cortex in processing AF during piano performance (Maidhof et al., 2010; Pfordresher et al., 2014) or sensorimotor sequence learning (Herrojo Ruiz et al., 2017). The limited sensitivity of the L2-norm MNE to this region might account for this apparent discrepancy. However, our reported sources associated with processing AF (i.e., STG) or differential encoding of boundary elements (DLPFC) might be more consistent with these processes.

## Conclusion

The present findings emphasize the importance of boundaries in sensorimotor sequence learning and extend previous studies by showing that the differential encoding of boundaries and within-sequence elements is sensitive to changes in auditory feedback and relies on dorsolateral prefrontal and higher-level motor areas. This finding is particularly relevant for understanding the neural circuitry behind the encoding of serial order position of actions in behavioral sequences that rely on auditory-motor coupling. Thus, it has implications for future studies investigating sequence learning in music, speech and singing.

## Author Contributions

GM was involved in the data analysis and the writing of the manuscript. MHR was involved in the design of the study, data acquisition, data analysis and the writing of the manuscript. VVN, GC and BM were involved in the design of the study and a critical revision of the manuscript.

## Conflict of Interest Statement

The authors declare that the research was conducted in the absence of any commercial or financial relationships that could be construed as a potential conflict of interest.

## Footnotes

^1^http://surfer.nmr.mgh.harvard.edu/

^2^www.martinos.org/mne/

## Supplementary Material

FIGURE S1 The effect of altered feedback (AF) on the timing performance of the subsequent keystroke. The plot shows the timing performance (mean IOI, ms) at the subsequent keystroke, +1, after normal feedback (NF; gray) or altered feedback (AF; black) at each element of the 4-note sequences (Position 1, 2, 3 and 4). When participants heard altered feedback, there was a post-feedback slowing at the next keystroke, which was prominent only after AF on boundary elements (significant for the Position 4, *p < pthr* = 0.02, PS_dep_ = 0.65; trend relative to *pthr* for the Position 1, *p* = 0.06, PS_dep_ = 0.70). **p* < pthr, non-parametric permutation test, pthr estimated to correct for multiple comparisons, see “Materials and Methods” section in the main text.
