# Supplementary figures and images for: Disruption of Boundary Encoding During Sensorimotor Sequence Learning: An MEG Study
Source: Front Hum Neurosci. 2018 Jun 12;12:240. doi: 10.3389/fnhum.2018.00240 (PMC6005865; doi:10.3389/fnhum.2018.00240)

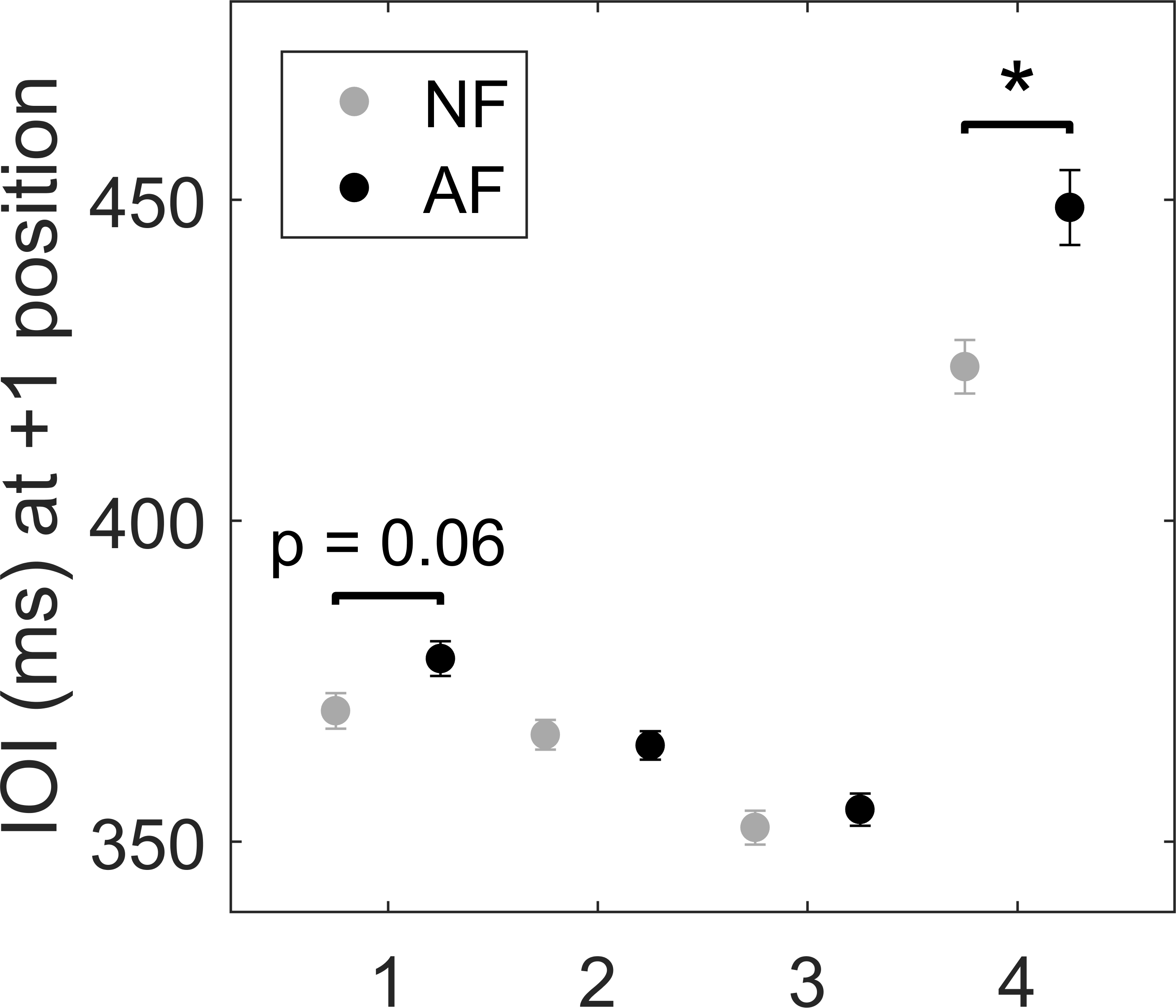

Supplement: FIGURE S1 — The effect of altered feedback (AF) on the timing performance of the subsequent keystroke. The plot shows the timing performance (mean IOI, ms) at the subsequent keystroke, +1, after normal feedback (NF; gray) or altered feedback (AF; black) at each element of the 4-note sequences (Position 1, 2, 3 and 4). When participants heard altered feedback, there was a post-feedback slowing at the next keystroke, which was prominent only after AF on boundary elements (significant for the Position 4, p < pthr = 0.02, PSdep = 0.65; trend relative to pthr for the Position 1, p = 0.06, PSdep = 0.70). *p < pthr, non-parametric permutation test, pthr estimated to correct for multiple comparisons, see “Materials and Methods” section in the main text. [file Image_1.TIF]
